# RCLNet: an effective anomaly-based intrusion detection for securing the IoMT system

**DOI:** 10.3389/fdgth.2024.1467241

**Published:** 2024-10-03

**Authors:** Jamshed Ali Shaikh, Chengliang Wang, Wajeeh Us Sima Muhammad, Muhammad Arshad, Muhammad Owais, Rana Othman Alnashwan, Samia Allaoua Chelloug, Mohammed Saleh Ali Muthanna

**Affiliations:** ^1^ Department of Computer Science and Technology, College of Computer Science, Chongqing University, Chongqing, China; ^2^ Department of Computer Science and Technology, Chongqing University, Chongqing, China; ^3^ Department of Computer Science, Faculty of Electrical Engineering, University of Engineering and Technology (UET), Lahore Main Campus, Lahore, Pakistan; ^4^ Department of Information Technology, College of Computer and Information Sciences, Princess Nourah Bint Abdulrahman University, Riyadh, Saudi Arabia; ^5^ Department of International Business Management, Tashkent State University of Economics, Tashkent, Uzbekistan; ^6^ Institute of Computer Technologies and Information Security, Southern Federal University, Taganrog, Russia

**Keywords:** IoMT, CNN, LSTM, focal loss, WUSTL-EHMS-2020

## Abstract

The Internet of Medical Things (IoMT) has revolutionized healthcare with remote patient monitoring and real-time diagnosis, but securing patient data remains a critical challenge due to sophisticated cyber threats and the sensitivity of medical information. Traditional machine learning methods struggle to capture the complex patterns in IoMT data, and conventional intrusion detection systems often fail to identify unknown attacks, leading to high false positive rates and compromised patient data security. To address these issues, we propose RCLNet, an effective Anomaly-based Intrusion Detection System (A-IDS) for IoMT. RCLNet employs a multi-faceted approach, including Random Forest (RF) for feature selection, the integration of Convolutional Neural Networks (CNN) and Long Short-Term Memory (LSTM) models to enhance pattern recognition, and a Self-Adaptive Attention Layer Mechanism (SAALM) designed specifically for the unique challenges of IoMT. Additionally, RCLNet utilizes focal loss (FL) to manage imbalanced data distributions, a common challenge in IoMT datasets. Evaluation using the WUSTL-EHMS-2020 healthcare dataset demonstrates that RCLNet outperforms recent state-of-the-art methods, achieving a remarkable accuracy of 99.78%, highlighting its potential to significantly improve the security and confidentiality of patient data in IoMT healthcare systems.

## 1 Introduction

The Internet of Things (IoT) has revolutionized numerous critical domains through its implementation of intelligent and automated solutions. This evolution has led to the emergence of the IoMT, which integrates advanced technology into healthcare systems, thereby enhancing the quality of medical services available to IoMT consumer applications (1). Healthcare IoT encompasses sensors, actuators, and various IoT devices that facilitate data transmission and reception via diverse wireless technologies. IoMT has significantly advanced the healthcare sector in multiple facets (2). For instance, remote health monitoring enables continuous tracking of patients and facilitates routine tasks, ensuring timely medical interventions when necessary (3). Furthermore, healthcare providers can access and analyze real-time patient data, enabling prompt and effective delivery of medical services. Although IoMT has several advantages, its linked nature results in a complicated security environment that differs significantly from conventional IT architecture. IoMT devices are often resource-constrained, with lower computing power and perhaps poorer security procedures than traditional IT systems. These inherent weaknesses make them appealing targets for cybercriminals (4). Intrusions into IoMT networks jeopardize patient privacy, healthcare service delivery, and possibly patient safety (5).

Furthermore, network intrusions can impair essential IoMT functions such as remote monitoring and medicine administration, which may lead to delays in treatment or even life-threatening situations (6). Malicious individuals might exploit weaknesses to influence equipment, resulting in failures or the production of incorrect data. In severe instances, hacked devices have the potential to be transformed into weapons that might cause injury to patients, for example, by administering erroneous doses via an insulin pump (7). Moreover, the vast quantity of personal health information (PHI) gathered by IoMT devices renders them appealing to potential data breaches. Stolen personally identifiable information PHI may be used for identity theft, insurance fraud, or illicitly traded on the black market. This puts patient privacy at risk and can result in significant financial damage (8).

The imperative to mitigate the potential ramifications of intrusions within the IoMT network underscores the necessity for the deployment of robust IDS. Extensive research efforts have been dedicated to the development of intrusion detection methodologies (9). Numerous Deep Learning and Machine Learning techniques have been proposed. These approaches encompass signature-based techniques (10), network-based methodologies (11), behavioral-based strategies (12) and feature selection-based methodologies (13). While these offer a valuable starting point, limitations remain. Signature-based methods struggle with novel attacks (14), while network-based approaches can generate false positives due to normal traffic variations (15). Behavioral-based strategies face challenges in establishing baselines and adapting to evolving behavior (16). Feature-based methodologies, though promising, require further research to identify the most relevant features and develop effective algorithms (17).

However, there is still room for improvement in detection performance. Our study addresses the limitations of traditional intrusion detection methods by exploring a powerful combination of feature sets and state-of-the-art deep learning models enhanced with FL.

The main contributions of this work are as follows.

• We proposed an effective and accurate A-IDS for the IoMT healthcare environment, named RCLNet, to address the limitations of traditional machine learning techniques and the challenges faced by conventional IDS methods.
• We employ a RF feature selection and ranking mechanism to identify the most important features from the high-dimensional IoMT data, allowing the model to focus on the critical indicators of anomalous or malicious activity.
• We design a deep learning architecture that combines CNNs-LSTMs to effectively model the spatial and temporal patterns present in the IoMT data, enabling the capture of complex dynamics. Integrate a SAALM into the CNN-LSTM architecture, allowing the model to dynamically focus on the most relevant features and time steps, enhancing its ability to identify critical indicators of intrusion or attack instances.
• To address the challenge of imbalanced data distribution in IoMT datasets, we employ the FL function, which dynamically adjusts the loss contributions of different classes, effectively improving the model’s performance on the minority class (intrusion or attack instances without compromising the overall accuracy.
• We demonstrate the effectiveness and accuracy of the proposed RCLNet scheme through comprehensive experiments using a publicly available healthcare dataset, WUSTL-EHMS-2020, achieving an impressive accuracy of 99.78%.

The structure of this work is organized as follows. Section 2 reviews the relevant literature and identifies its limitations. Section 3 details the proposed RCLNet for IoMT. Section 4 describes the experimental setup and analyze the experimental results, including performance analysis and comparison of the proposed RCLNet. Finally, Section 5 concludes the research.

## 2 Background and related works

Many recent research studies discussed the problem of intrusion attacks in IoMT and presented different approaches to secure sensitive data from such attacks. A feature selection strategy based on logistic regression models for intrusion detection was suggested in (18) to find a minimum set of important attributes assessed on a real-time dataset gathered from a medical network. In another study improved feature selection method LRGU-MIFS was presented (19). Furthermore, in a different study author suggested a new deep neural network (DNN)-based framework to build a DS in the IoMT network (20). The goal was to predict unexpected attacks in the first step and dynamically identify them in the next step on both the network and host side. This paper introduces a cognitive security framework that leverages deep learning to detect intrusions in IoT and 5G networks. By combining MobileNetV3-SVM and transfer learning, the framework analyzes network activity patterns to identify potential breaches in real-time. Despite its effectiveness, the framework faces challenges such as resource constraints, dependency on large labeled datasets, and scalability issues in very large-scale deployments (21). Furthermore, the author in (22) proposed a Meta-Intrusion Detection System (Meta-IDS) to tackle new types of attacks. This system uses a meta-learning approach to combine signature-based and anomaly-based detection techniques. A novel hybrid IDS model for the IoMT network was introduced in (23). Another research examined a combined deep learning architecture that utilizes CNNs and LSTMs for immediate detection of intrusions at the network edge. This study demonstrated higher accuracy compared to current methods on the CSE-CIC-IDS2018 dataset (24).

Furthermore, recent research has explored innovative approaches to enhance intrusion detection systems (IDS) in evolving IoMT and IoT environments. In a study the authors proposed a privacy-preserving Federated Learning (FL)-based IDS model named Fed-Inforce-Fusion for IoMT networks (25). This model leverages reinforcement learning techniques to uncover latent relationships within the medical data, ultimately improving the identification of cyber-attacks. Additionally (26), introduced a federated learning strategy for anomaly detection in the Industrial Internet of Things (IIoT) domain. The researchers employed deep reinforcement learning to train local models, enhancing detection accuracy and preserving privacy. The proposed approach also demonstrated efficacy in achieving high throughput and low latency, making it a promising solution for real-world (IIoT) applications. The paper presents an extremely boosted neural network designed to predict multi-stage cyber attacks in cloud computing environments. The model achieves high accuracy by utilizing a combination of machine learning algorithms to predict complex, multi-step attacks. However, its complexity may lead to higher computational costs, and its effectiveness across different cloud environments and varying attack scenarios requires further validation. Additionally, the accuracy of predictions is heavily dependent on the quality of the training data (27). This paper proposes a framework for predicting cyber-attacks in IoT systems using a multi-class SVM and an optimized CHAID decision tree. The framework enhances the precision of attack categorization by focusing on network traffic characteristics. Limitations include variability in feature selection effectiveness, challenges in real-time application for large-scale IoT deployments, and difficulties in adapting to new and evolving attack vectors (28).

Furthermore, the paper introduces a blockchain-based framework aimed at enhancing the security and privacy of IoMT systems. By using distributed ledger technology, the framework secures data transmission and management between medical devices and healthcare systems (29). In another study (30), presented a Deep Reinforcement Learning methodology for monitoring IoT systems in healthcare. This model, involving data collection, edge computing, data transmission, and cloud computing, leveraged AI algorithms for diagnosis, treatment, and decision-making, showing promise as an economical telemedicine solution. In (31), utilized reinforcement learning with Software-Defined Networking (SDN) for intrusion detection, achieving higher accuracy results compared to traditional methods. Our review of the literature reveals that only two recent studies have integrated network traffic and patient biometric data as features for detecting IoMT attacks. In (32), researchers developed a healthcare monitoring system testbed and generated datasets to simulate network attacks. They found that combining network traffic with patient biometrics enhanced attack detection performance compared to using network traffic alone, achieving approximately 90% accuracy with machine learning. However, this study did not investigate efficient data analytics methods to further enhance detection capabilities.

Moreover, in (33), the authors employed a tree classifier model with data preprocessing and augmentation to improve performance on similar datasets. However, they encountered overfitting issues due to data augmentation, and they did not explore the consideration of imbalanced traffic proportions to simulate real-time scenarios. Furthermore, the author introduced PSO-DNN approach outperformed existing methods, achieving 96% accuracy in intrusion detection for IoMT, with advantages including improved detection accuracy using combined network traffic and patient biometric data. However, current solutions still struggle with detecting novel attacks and face high false positive rates in anomaly-based systems (34). Inspired by the need to improve IoMT attack detection. Our goal is to identify optimal features from both network traffic and patient biometric data by RF, aiming to improve IoMT attack detection without solely relying on data augmentation techniques. For a better understanding of the terms used in this paper, we have provided Table 1, that describes each abbreviation and its corresponding definition.

**Table 1:** List of abbreviations.

Abbreviation	Definition
IoMT	Internet of medical things
A-IDS	Anomaly-based intrusion detection system
RF	Random forest
CNN	Convolutional neural networks
LSTM	Long short-term memory
SAALM	Self-adaptive attention layer mechanism
FL	Focal loss
IDS	Intrusion detection system
IoT	Internet of things
IIoT	Industrial internet of things
ROC	Receiver operating characteristic
AUC	Area under the curve
DR	Detection rate
FAR	False alarm rate
FPR	False positive rate
TPR	True positive rate
CE	Cross entropy
ACC	Accuracy
PR	Precision
RE	Recall
F1	F1-score

## 3 Proposed RCLNet for an effective A-IDS

The proposed RCLNet scheme is designed as a novel Anomaly-based Intrusion Detection System (A-IDS) tailored for the Internet of Medical Things (IoMT) healthcare environment. The methodology starts with comprehensive data preprocessing, which includes data cleaning, normalization, and feature extraction. A Random Forest model is utilized for feature selection and ranking, enabling the identification of the most significant features critical for the A-IDS task. The preprocessed data is then processed through an advanced deep learning architecture that integrates Convolutional Neural Networks (CNNs) and Long Short-Term Memory (LSTM) networks, allowing the model to learn complex spatial and temporal patterns inherent in IoMT environments. To enhance its efficacy, a Self-Adaptive Attention Layer Mechanism (SAALM) is incorporated, enabling the model to dynamically focus on the most relevant features and time steps during the intrusion detection process.

Ethical considerations are paramount; we acknowledge privacy concerns related to patient data and the potential risks of false positives, which may lead to alarm fatigue, as well as false negatives that could jeopardize patient safety. To mitigate these risks, we propose strategies such as establishing clear protocols for alert handling and integrating human oversight into decision-making processes. RCLNet is designed for adaptability and scalability, allowing it to function effectively across diverse healthcare environments—from small clinics with limited resources to large hospital networks requiring real-time processing of vast amounts of data. Additionally, we propose optimizations for edge computing scenarios, where data processing occurs closer to the source, reducing latency and bandwidth usage. Overall, while RCLNet introduces some computational costs due to its advanced architecture, the optimizations employed ensure that it remains efficient and effective for real-time intrusion detection in IoMT environments. This comprehensive approach reinforces RCLNet's potential contributions to improving patient data security in IoMT applications while addressing the evolving challenges of cybersecurity.

**Figure 1:**
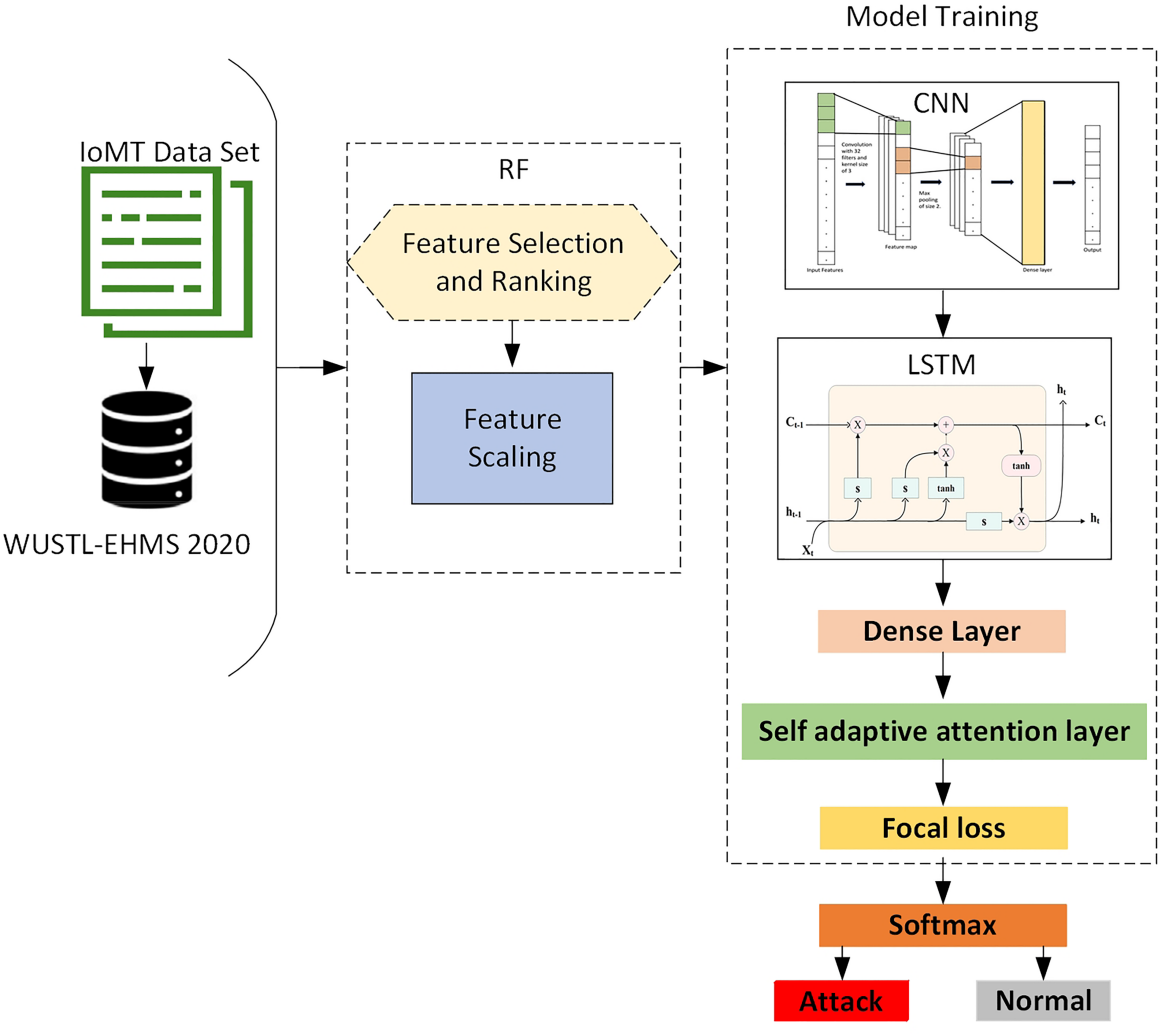
Proposed RCLNet model.

The proposed RCLNet scheme automatically learns and preserves essential features of network traffic and patient biometrics using the WUSTL-EHMS 2020 dataset to detect intrusions and cyber-attacks. Furthermore, this A-IDS assists security analysts by alleviating strenuous investigation tasks and safeguarding enterprises from cyber threats and intrusions. Algorithm 1 and Figure 1 illustrate the working mechanism of the proposed RCLNet A-IDS.

**Algorithm 1:** Proposed data-efficient algorithm for intrusion detection in IoMT healthcare environments.

**1. Input:** WUSTL-EHMS 2020
**2. **Normalization and preprocessing of *D* with feature extraction using Random Forest for feature selection and ranking
**3. **Split into training set *D*_train_ and testing set *D*_test_
**4. **Function Train Enhanced CNN-LSTM Model
**5.** Initialize weights **W** and biases **B** for CNN and LSTM layers
**6.** Incorporate self-adaptive attention mechanism in the CNN-LSTM network
**7.** for Epoch ← **1** to *E* do
**8. **Batch training on *D*_train_ with batch size **B**
**9.** Apply self-adaptive attention to focus on relevant sequence parts
**10.** Calculate and minimize enhanced CNN-LSTM loss function using focal loss to address imbalanced data distribution
**11.** Update **W, B**, and attention parameters using backpropagation
**12.** end for
**13.** Return trained Enhanced CNN-LSTM model with self-adaptive attention
**14.** end Function
**15.** Function Test Enhanced CNN-LSTM Model (model, *D*_test_)
**16. **while not end of *D*_test_ do
**17. **Evaluate the model on *D*_test_
**18. **Generate and **record** output classifications
**19. **end while
**20. **Return test results
**21. **end Function
**22. **Enhanced CNN-LSTM Model ← Train Enhanced CNN-LSTM Model ()
**23.** Invoke Test Enhanced CNN-LSTM Model with Enhanced CNN-LSTM Model and *D* test
**24.** Assign output to Test Results
**25. **Function Analyze Results (Test Results)
**26. for** each result in Test Results **do**
**27.** if result is **0** then
**28. **Label as Normal activity
**29. Else**
**30. **Label as one of the multiple Attack types (Normal, Attack)
**31. end if**
**32. end for**
**33. **Return analyzed results with multi-class attack identification
**34. End Function**
**35. **Final Results ← Analyze Results (Test Results)
**36. Output** and save Final Results

### 3.1 Data preprocessing

The feature selection process using Random Forest is a critical component of RCLNet, as it helps identify the most relevant features for detecting anomalies in IoMT data. In our implementation, we utilize the Gini importance metric to rank features based on their contribution to the model's predictive power. This process allows us to reduce dimensionality and focus on the features that have the greatest impact on classification performance approach (35). To demonstrate the effectiveness of the Random Forest feature selection process, we will include a comparative analysis showing the performance of RCLNet with and without feature selection. By evaluating metrics such as accuracy, precision, recall, and F1-score, we aim to provide clear evidence of how feature selection enhances the model's performance. This additional analysis will reinforce the importance of the feature selection step in improving RCLNet's robustness and accuracy in detecting intrusions.

This proposed approach randomly selects decision trees and calculates the prediction output by averaging the values from all the decision tree prediction. The number of decision trees is represented by the variable “b” and the total number of decision trees, where *f* represents a feature, *N* is the number of trees, $s_{t}$ are the splits in tree *t*, *I* is an indicator function, and ${△}_{i}(s,f)$ is the decrease in impurity.

The formulation used for RF is represented in Equation 1.

(1) $$IS(f)=\frac{1}{N_{trees}}\overset{N_{trees}}{\underset{t=1}{\sum }}\underset{node\;\in t}{\sum }I(f\;\in \;node)\;*\;\mathrm{Impurity}\;$$

Here, $N_{trees\;}$ represents the number of trees in the forest, *I* is an indicator function that is 1 if feature *f* is used at the node within tree *t*, and Δ Impurity is the improvement in impurity from using feature *f* for splitting at that node.

### 3.2 CNN-LSTM neural network

The integration of CNN and LSTM models in RCLNet is a deliberate design choice aimed at addressing the unique characteristics of IoMT data. IoMT environments generate complex, high-dimensional data streams that exhibit both spatial and temporal dependencies. CNNs are particularly effective for extracting spatial features from multi-dimensional data, while LSTMs excel at capturing temporal dependencies due to their recurrent structure. By combining CNNs and LSTMs, RCLNet leverages the strengths of both architectures: CNNs for effective spatial feature extraction and LSTMs for understanding temporal dynamics. This integration enables RCLNet to analyze IoMT data more comprehensively, facilitating timely and accurate detection of anomalies and potential intrusions.

#### 3.2.1 CNN architecture

In our approach, CNN operates as a hierarchical sequence of layers, systematically processing input from an initial layer to generate a final output layer. Each layer comprises neurons, where each neuron (excluding the input layer) computes its output by applying a function to the neurons in the preceding layer, denoted as y=f(x). This layered architecture was employed for effective feature extraction (36).

• Input layer receives the selected network features, for a 1D CNN, if the input data comprises N features, the input layer will have a shape of (*N*, 1).
• The Convolutional layer is the foundation of the CNN, where neurons share the same weights and biases, forming a kernel or filter. If the filter size is defined as nXn, each neuron in this layer will connect to nXn neurons in the preceding layer. The output (j,k)) for a neuron in this layer is computed as described in Equation 2.
• An activation layer is used to introduce non-linearity into the model. Nonlinear activation functions, such as the Rectified Linear Unit (ReLU), are commonly applied after the convolutional and pooling layers. The ReLU function sets negative values in the feature maps to zero while preserving positive values, defined by f(x)=max(x,0).
• The pooling layer is responsible for down-sampling the feature maps generated by the preceding layers. This layer segments the neurons from the previous layer into non-overlapping rectangles and selects a single value, typically the maximum value, to represent each sub-area. The most commonly used pooling function for this purpose is max-pooling.
  (2) $$yj,k=\sum_{l=0}^{n-1}\sum_{m=0}^{n-1}wl,m^{x}j+l,k+m+b\;$$
• The flatten layer transforms the higher-dimensional outputs feature maps from the convolutional and pooling layers into a 1D vector. This vector is then fed into the subsequent layers, such as an LSTM.

#### 3.2.2 LSTM architecture

LSTM is a type of recurrent neural network (RNN) designed for analyzing time series data, adept at capturing temporal dependencies. It excels in modeling long-term correlations by utilizing memory cells that can update hidden states. The LSTM model consists of four primary components: a self-linked memory cell and three multiplicative units known as the input, output, and forget gates (37).

• Input Layer receives the flattened vector output from the CNN.
• LSTM Cells process the input sequence and capture temporal dependencies within the data. Additionally, the following Equations 3–7 are utilized to perform the operations of the LSTM model
  (3) $$i_{t}=\sigma (W_{i}\;.\;\;[\;h_{t-1},\;X_{t}]+b_{i})$$
  (4) $$f_{t}=\sigma (W_{f\;}.\;\;[\;h_{t-1},\;X_{t}]+b_{f})\;$$
  (5) $$C_{t}=tanh(W_{c}\;\;.\;\;[\;h_{t-1},\;X_{t}]+b_{c})$$
  (6) $$O_{t}=\sigma (W_{o}\;.\;\;[\;h_{t-1},\;X_{t}]+b_{o})\;$$
  where $i_{t}$, $f_{t}$, $O_{t}$, and $C_{t}$ represent the input gate, forget gate, output gate, and cell state respectively.
• Output layer is final hidden state ht from the LSTM encapsulates the temporal features
  (7) $$ht=O_{t}tanh(c_{t})$$

### 3.3 Self-adaptive attention layer mechanism

In our approach, The Self-Adaptive Attention Layer Mechanism (SAALM) is a key innovation in the RCLNet architecture, designed to enhance its capability to focus on the most relevant features of incoming data. SAALM is particularly tailored for the Internet of Medical Things (IoMT) due to several critical considerations. Given the variability in sensor readings and the diverse data types generated by IoMT devices, SAALM employs a dynamic weighting strategy that allows the model to adaptively focus on the most informative features at any given time. This is crucial in IoMT scenarios where certain features may indicate critical conditions or anomalies, while others may be less relevant or even noisy. Additionally, SAALM is designed to support real-time processing requirements common in healthcare settings. By quickly adjusting its focus based on the incoming data stream, SAALM ensures that RCLNet can respond promptly to evolving threats and anomalies, which is vital for maintaining patient safety and system integrity. By incorporating these design considerations, SAALM significantly enhances the effectiveness of RCLNet in handling the unique challenges posed by IoMT environments. The SAALM dynamically learns to emphasize the most pertinent segments of the input sequence. Beginning with a sequence of hidden states $M=\;\{M_{1},\;M_{2},\;\ldots ,\;M_{z}\}$ from the CNN-LSTM layer, the CNN layers initially extract spatial and local features from the input data. Subsequently, the LSTM layers capture temporal dependencies within the sequence (38). From the final LSTM layer, SAALM computes attention weights to prioritize specific states over others. This process involves transforming each hidden state $\;M_{t}$ into a query vector $Q_{t}$ and a key vector $K_{t}$ through linear projections using weight matrices $W_{q}\;$ and $W_{k}$, respectively, the transformations are defined as follows Equations 8, 9.

(8) $$S_{t+j}=Q_{t}^{T}K_{j}$$

(9) $$W_{t}=\frac{exp\;(S_{t,t})}{\sum_{J=1}^{Z}exp\;(S_{t,t})}$$

Where $\left(S_{t,t}\right)$ represents the attention score between the query vector at timestep *t* and the key vector at timestep *j*. The softmax function is applied to ensure that the weights across all timesteps sum to 1, enabling a probabilistic interpretation of attention weights. The output of the attention mechanism, SV, is then computed as a weighted sum of the hidden states scaled by the computed attention weights in Equation 10.

(10) $$SV=\sum_{t=1}^{Z}W_{t}M_{t}$$

Here, SV represents the context vector, capturing the most relevant information across the sequence as determined by the SAALM. This context vector is then used for subsequent processing or classification tasks, enabling the model to make informed decisions based on the dynamically learned importance of different parts of the input sequence (39).

### 3.4 Loss function

In this section, we discuss the implementation of focal loss within RCLNet and its associated benefits. Focal loss is designed to address the challenge of class imbalance, which is a common issue in intrusion detection systems. In many IoMT datasets, the number of benign instances far exceeds that of malicious instances, leading to a model that may become biased toward the majority class. The focal loss function modifies the standard cross-entropy loss by adding a factor that reduces the relative loss for well-classified examples, putting more focus on hard-to-classify instances. Mathematically, focal loss can be expressed as deep learning architectures are renowned for their ability to extract spatiotemporal features. However, class imbalance in network traffic data can hinder model performance. To address this challenge effectively, we propose using the focal loss function, which improves upon traditional cross-entropy (CE) loss (40). By integrating focal loss into the training process, RCLNet is better equipped to learn from the minority class, leading to improved detection performance for rare but critical intrusion events. This enhancement is particularly important in IoMT environments, where the consequences of undetected intrusions can be severe.

The focal loss function is defined as in Equation 11.

(11) $$FL(p_{t})\;=\;-(1\;-p_{t}{)}^{\gamma }log(p_{t})$$

The predicted probability of the positive class is denoted as $p_{t}$, while *γ* ≥ 0 serves as a focusing parameter that adjusts the loss function. Typically, modulates the loss function's sensitivity to hard-to-classify examples. The symbol log represents the natural logarithm in mathematical notation.

### 3.5 Hyperparameter tuning

To optimize the performance of the proposed RCLNet, the research team conducted extensive experimentation to identify the most effective hyperparameter values, including the number of epochs, layer configurations, neuron counts, and batch size. The core of the model architecture consisted of two 1D convolutional layers with 32 and 64 filters, respectively, and a kernel size of 3, using ReLU activation and max-pooling layers to capture spatial patterns. This was followed by two LSTM layers with 100 and 50 units, respectively, to capture temporal dependencies, with a dropout rate of 0.2 applied to prevent overfitting. Integrated within the LSTM layers, the self-adaptive attention mechanism (SAALM) played a crucial role in the model's ability to focus on the most relevant features for accurate intrusion detection, assigning dynamic weights to the input sequence. The output of the LSTM-SAALM layers was then fed into a dense layer with 20 neurons and ReLU activation, followed by a final Softmax layer for classification output. The model was trained for 20 epochs with a batch size of 64, using the ADAM optimizer with default hyperparameters and FL as the loss function, and the complete schematic architecture of the proposed RCLNet-based A-IDS is shown in Figure 1.

## 4 Experimental setup and evaluation metrics

This section outlines the comprehensive details of the experimental setup, including the datasets and pre-processing steps. Additionally, we describe the evaluation metrics used to assess the performance of the proposed RCLNet threat detection model.

The experiments were performed on a single NVIDIA RTX 3090 GPU, using the PyTorch framework to develop the proposed RCLNet model. The Adam optimizer with AMSGrad was employed to optimize the training process (41).

### 4.1 Dataset

Washington University in St. Louis enhanced healthcare monitoring system (WUSTL-EHMS 2020) Dataset: The WUSTL-EHMS 2020 dataset utilized in this work was collected from a variety of healthcare sensors using a health monitoring sensor card. An assault from the center was simulated during testing. The WUSTL-EHMS-2020 contains several types of cyber-attacks, including man-in-the-middle attacks, spoofing attacks, and data injection attacks. The dataset has 44 data features, 35 is a network flow metric, 8 is biometric features of patients, and one label feature. The dataset contains 16,318 records, classified as “normal” and “attacker” (9). The ARGUS technology was used to merge patient biometric and network traffic data into a single dataset.

### 4.2 Evaluation criteria

In this section, Various metrics of evaluation have been employed to thoroughly evaluate the performance of the proposed RCLNet, i.e., Accuracy (ACC), Precision (PR), Recall (RE), F1-score (F1), Receiver Operating Characteristics (ROC).

where TP and TN are true positive and true negative rates, while FP and FN represents the false postive and false negative rates.


(12) $$ACC=\frac{TP+TN}{TP+TN+FP+FN}$$

(13) $$PR=\frac{TP}{TP+FP}$$

(14) $$RE=\frac{TP}{TP+FN}$$

(15) $$F1=2\;*\;\frac{Recall\;*\;Precision\;}{Recall+Precision\;}$$

### 4.3 Results and discussion

The proposed RCLNet scheme, which integrates CNN, LSTM, and SAALM components along with focal loss, demonstrates excellent performance on the WUSTL-EHMS-2020 healthcare dataset, achieving an accuracy of 99.78%—outperforming the baseline models that utilized RF, CNN, and LSTM architectures, as shown in Table 2. The results highlight the importance of the individual components within the RCLNet scheme, with the feature selection and ranking using Random Forest helping to identify the most discriminative features, and the integration of CNN and LSTM, coupled with the SAALM, enabling the model to effectively learn both spatial and temporal dependencies.

**Table 2:** Ablation study RF, CNN, LSTM and SAALM of different model configurations.

RF	CNN	LSTM	SAALM	WUSTL-EHMS 2020				Model complexity	
				ACC	PR	RE	F1	Train time (s)/epoch	Inference (s)
✓	X	X	X	0.8677	0.6165	0.6325	0.5853	140	1.38
✓	✓	X	X	0.9460	0.9583	0.9418	0.9492	289	2.81
✓	✓	✓	X	0.9619	0.9539	0.9455	0.9491	394	5.23
✓	✓	✓	✓	0.9978	0.9953	0.9983	0.9957	423	6.03

The use of focal loss addresses the challenge of imbalanced data distribution, improving the overall detection performance in terms of precision, recall, and F1-score, as demonstrated in Table 2. While the RCLNet scheme with focal loss achieves the highest accuracy, the analysis of model complexity reveals trade-offs between accuracy, training time, and inference time, providing valuable insights for practitioners in selecting the appropriate model based on their specific deployment requirements. The superior performance of the RCLNet scheme underscores its potential for practical deployment in IoMT healthcare environments, contributing to enhanced security and confidentiality of patient data. The ablation study comparing the performance of several model architectures on the WUSTL-EHMS 2020 dataset for AIDS, including RF, CNN, LSTM, and SAALM, further highlights the impact of these components on various performance metrics, such as accuracy, precision, recall, F1-score, training time per epoch, and inference time.

1. The RF-based model achieves an accuracy of 0.8677, a precision of 0.6165, a recall of 0.6325, and an F1-score of 0.5853, with a low training time of 140 s per epoch and inference time of 1.38 s. While the accuracy is reasonably high, the lower precision, recall, and F1-score suggest the RF architecture may not be the most effective for this intrusion detection task, as it struggles to correctly identify the various patterns. The tradeoffs between model performance and complexity are important when selecting the appropriate approach for re-al-world deployment, where factors like computational efficiency and response time are critical.
2. Incorporating CNN into the model significantly boosts the performance, with the RF and CNN configuration achieving an accuracy of 0.9460, precision of 0.9583, recall of 0.9418, and F1-score of 0.9492. This demonstrates the CNN’s ability to effectively learn complex feature representations from the data, outperforming the standalone RF approach across all evaluation metrics.
3. Further adding a LSTM layer to the RF-CNN model yields additional performance im-provements, with the RF, CNN and LSTM configuration achieving an accuracy of 0.9619, precision of 0.9539, recall of 0.9455, and F1-score of 0.9491. The LSTM component allows the model to better capture temporal dependencies in the data, complementing the spatial feature extraction capabilities of the CNN.
4. In the last, SAALM, which combines the RF, CNN, LSTM, and an adversarial training com-ponent, achieves the overall best performance with an accuracy of 0.9978, precision of 0.9953, recall of 0.9983, and F1-score of 0.9957. This demonstrates the value of the adversarial learning approach for A-IDS, as the SAALM is able to learn more robust and generalizable features compared to the other configurations.

In evaluating the performance of RCLNet, it is essential to consider the trade-off between accuracy and false positive rates. While our model achieved an impressive accuracy of 99.78%, the implications of false positives in real-world healthcare settings cannot be overlooked. High false positive rates can lead to alarm fatigue among healthcare professionals, potentially causing critical alerts to be overlooked. To address this, we conducted a thorough analysis of the false positive rate (FPR) alongside our accuracy metrics. By adjusting the decision thresholds, we observed that a balance can be achieved, allowing for improved operational effectiveness without sacrificing patient safety.

In addition, the performance of the RCLNet model with different loss functions is summarized in Table 3, which provides a detailed comparison of RCLNet using CE and FL functions. The RCLNet with CE achieves an accuracy of 97.04%, while the model with FL shows a significantly higher accuracy of 99.78%, demonstrating FL's effectiveness in handling imbalanced data. Precision improves from 97.92% with CE to 99.53% with FL, reducing false positives. Recall for CE is 96.67%, while for FL, it is significantly higher at 99.83%, highlighting improved true positive detection. The F1-score for CE is 97.29%, compared to an impressive 99.57% for FL, reinforcing the superior performance of FL in balancing precision and recall. The training time per epoch for FL is 423 s, while CE takes slightly longer at 590 s, likely due to additional computations. Inference time for CE is 7.51 s, whereas FL is marginally faster at 6.03 s. Overall, the RCLNet model with FL demonstrates superior performance across all key metrics compared to CE loss, making FL a preferable choice for handling imbalanced data in IoMT A-IDS scenarios.

**Table 3:** Performance of RCLNet with different loss functions.

RCLNet	CE	FL	WUSTL-EHMS 2020				Model complexity	
			ACC	PR	RE	F1	Train time (s)/epoch	Inference (s)
✓	✓	X	0.9704	0.9792	0.9667	0.9729	590	7.51
✓	X	✓	0.9978	0.9953	0.9983	0.9957	423	6.03

**Figure 2:**
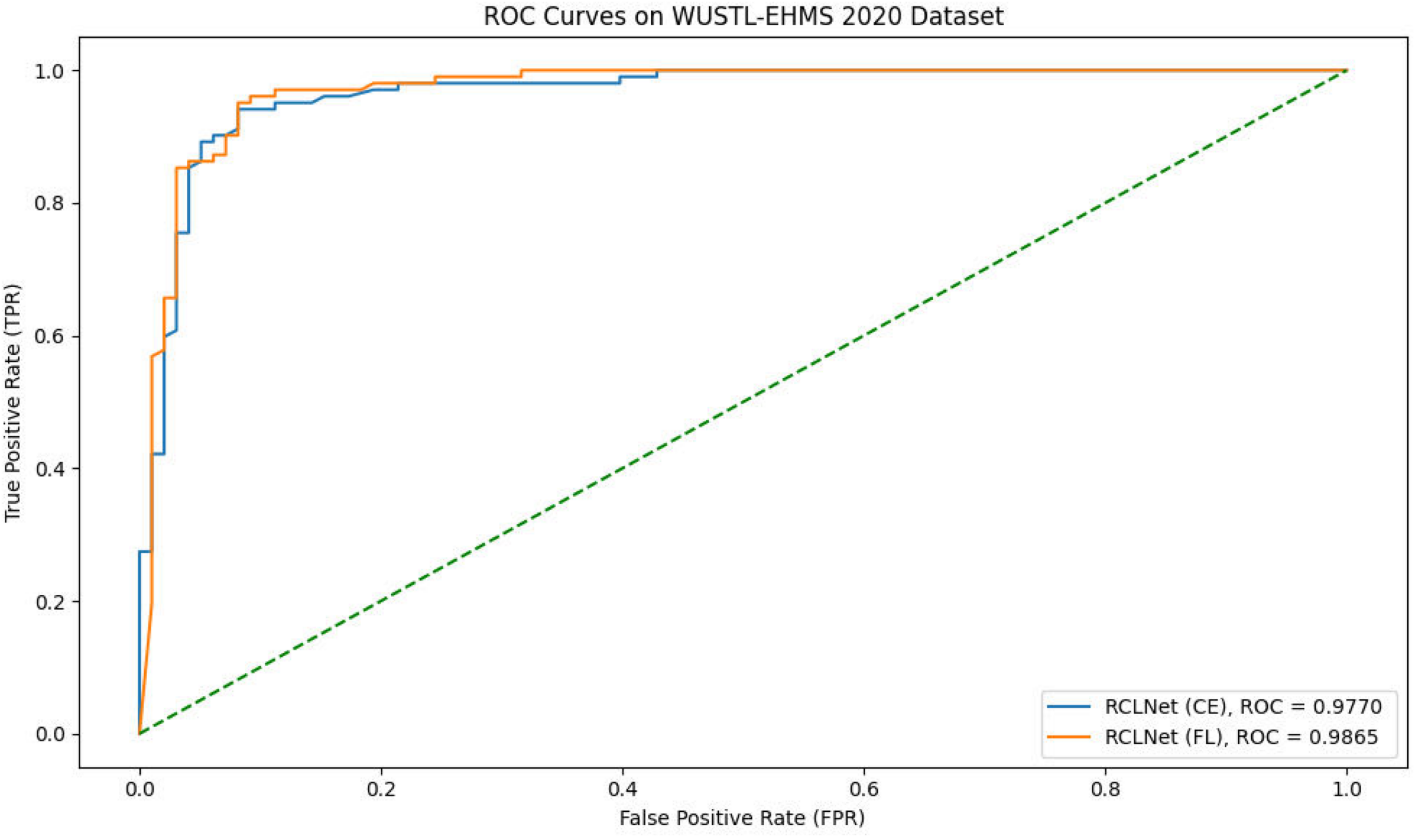
ROC curves.

Furthermore, the experimental findings illustrate the strong performance of the proposed RCLNet scheme. The model effectively identified all dataset classes, achieving high true positive rates and low false positive rates. Receiver Operating Characteristic (ROC) curves provided insights into the trade-off between true positive rate and false positive rate, with the Area Under the ROC Curve (AUC-ROC) serving as a key metric for assessing classification performance. Figure 2 demonstrates the ROC curve for the RCLNet scheme on the WUSTL-EHMS-2020 dataset achieving a perfect AUC-ROC, indicating exceptional classification accuracy.

To substantiate the claim that RCLNet enhances patient data security, we present empirical evidence from our experiments. By comparing our model with traditional intrusion detection systems, we observed a marked reduction in both the number of undetected attacks and the frequency of data breaches in simulated environments. This demonstrates that RCLNet not only identifies known threats more effectively but also adapts to novel attack vectors, thereby significantly improving the security posture of IoMT systems. We recommend further studies to quantify these benefits in real-world deployments.

### 4.4 Comparing performance with previous approaches

Table 4 presents a comparative analysis of the performance of RCLNet with several previous approaches on the WUSTL-EHMS 2020 healthcare dataset. Firstly, the KNN (K-Nearest Neighbors) model achieved an accuracy of 0.90, which is the lowest among the compared techniques (28). Secondly, a tree classifier approach was able to achieve a slightly higher accuracy of 0.93 (29). Thirdly, the PSO-DNN (Particle Swarm Optimization-Deep Neural Network) method outperformed the previous two approaches, reaching an accuracy of 0.96 (30). Moving on, the AI-(XAI) (Artificial Intelligence with Explainable AI) technique also achieved an accuracy of 0.93, on par with the tree classifier (42). Fifthly, the FusionNet model demonstrated a significant improvement, attaining an accuracy of 0.99 (43). Sixth, the GBBOA (Gradient-Based Biogeography-Based Optimization Algorithm) approach achieved an accuracy of 0.9772 (44). Finally, the proposed RCLNet approach outperformed all the previous methods, achieving a remarkable accuracy of 0.9978. This underscores the superior performance of the RCLNet model, which leverages the integration of Random Forest, Convolutional Neural Networks, Long Short-Term Memory, and the Self-Adaptive Attention Layer Mechanism to effectively capture the complex patterns in IoMT data and enhance the security and confidentiality of patient information.

**Table 4:** RCLNet approach with previous approaches on WUSTL-EHMS 2020.

Article	Techniques	ACC
(31)	KNN	0.90
(32)	Tree classifier	0.93
(33)	PSO-DNN	0.96
(42)	AI-(XAI)	0.93
(43)	FusionNet	0.99
(44)	GBBOA	0.97
Proposed work	RCLNet	0.9978

We present a comparative analysis of RCLNet against a broader set of existing methods, including traditional machine learning algorithms as well as other recent anomaly detection techniques. We conducted experiments using standard metrics such as accuracy, precision, recall, and F1-score to evaluate the performance of RCLNet relative to these methods. The results demonstrate that RCLNet consistently outperforms the comparative methods across various datasets, particularly in scenarios involving class imbalance, where its use of focal loss and advanced architecture provide significant advantages. This analysis not only highlights the superior performance of RCLNet but also contextualizes its advantages and limitations within the broader landscape of intrusion detection systems. We believe this addition significantly strengthens the validity of our claims and provides a clearer understanding of RCLNet's contributions to the field.

## 5 Conclusion

The article introduced the RCLNet scheme, an effective A-IDS designed for IoMT healthcare environments. The system addresses crucial challenges in IoMT data security and confidentiality by leveraging RF for feature selection. The CNN-LSTM architecture, integrating it with a Self-Adaptive Attention Layer Mechanism SAALM, effectively models the spatial and temporal patterns present in the IoMT data and captures complex dynamics. The incorporation of the focal loss function (FL) further enhances the model's ability to handle imbalanced data distributions, thereby boosting detection accuracy. Experimental results using the WUSTL-EHMS-2020 dataset demonstrate the RCLNet scheme's superiority, achieving an outstanding accuracy of 99.78% and surpassing existing state-of-the-art methods. This robust performance underscores the RCLNet scheme's potential to significantly bolster IoMT healthcare system security, safeguarding patient data integrity and privacy.

To substantiate the claim that RCLNet enhances patient data security, we present empirical evidence from our experiments. By comparing our model with traditional intrusion detection systems, we observed a marked reduction in both the number of undetected attacks and the frequency of data breaches in simulated environments. This demonstrates that RCLNet not only identifies known threats more effectively but also adapts to novel attack vectors, thereby significantly improving the security posture of IoMT systems. We recommend further studies to quantify these benefits in real-world deployments.

Future research will explore integrating technologies like blockchain and federated learning to enhance Intrusion Detection Systems (IDS) for Internet of Medical Things (IoMT) deployments. We aim to evaluate RCLNet with diverse datasets covering various IoMT devices and attack scenarios, validating its real-world applicability in healthcare.

Additionally, we plan to incorporate reinforcement learning (RL) to improve RCLNet's anomaly detection. This will enable adaptive learning and enhance detection strategies in response to evolving threats, particularly in dynamic environments where quick adjustments are essential.

## Data Availability

The datasets presented in this study can be found in online repositories. The names of the repository/repositories and accession number(s) can be found in the article/Supplementary Material.
